# Assessment of Emergency Medicine Resident Performance in an Adult Simulation Using a Multisource Feedback Approach

**DOI:** 10.5811/westjem.2018.12.39844

**Published:** 2018-12-17

**Authors:** Michael Jong, Nicole Elliott, Michael Nguyen, Terrence Goyke, Steven Johnson, Matthew Cook, Lisa Lindauer, Katie Best, Douglas Gernerd, Louis Morolla, Zachary Matuzsan, Bryan Kane

**Affiliations:** * Lehigh Valley Health Network, Department of Emergency and Hospital Medicine, Bethlehem, Pennsylvania; † University of South Florida Morsani College of Medicine, Tampa, Florida

## Abstract

**Introduction:**

The Accreditation Council for Graduate Medical Education (ACGME) specifically notes multisource feedback (MSF) as a recommended means of resident assessment in the emergency medicine (EM) Milestones. High-fidelity simulation is an environment wherein residents can receive MSF from various types of healthcare professionals. Previously, the Queen’s Simulation Assessment Tool (QSAT) has been validated for faculty to assess residents in five categories: assessment; diagnostic actions; therapeutic actions; interpersonal communication, and overall assessment. We sought to determine whether the QSAT could be used to provide MSF using a standardized simulation case.

**Methods:**

Prospectively after institutional review board approval, residents from a dual ACGME/osteopathic-approved postgraduate years (PGY) 1–4 EM residency were consented for participation. We developed a standardized resuscitation after overdose case with specific 1–5 Likert anchors used by the QSAT. A PGY 2–4 resident participated in the role of team leader, who completed a QSAT as self-assessment. The team consisted of a PGY-1 peer, an emergency medical services (EMS) provider, and a nurse. Two core faculty were present to administer the simulation case and assess. Demographics were gathered from all participants completing QSATs. We analyzed QSATs by each category and on cumulative score. Hypothesis testing was performed using intraclass correlation coefficients (ICC), with 95% confidence intervals. Interpretation of ICC results was based on previously published definitions.

**Results:**

We enrolled 34 team leader residents along with 34 nurses. A single PGY-1, a single EMS provider and two faculty were also enrolled. Faculty provided higher cumulative QSAT scores than the other sources of MSF. QSAT scores did not increase with team leader PGY level. ICC for inter-rater reliability for all sources of MSF was 0.754 (0.572–0.867). Removing the self-evaluation scores increased inter-rater reliability to 0.838 (0.733–0.910). There was lesser agreement between faculty and nurse evaluations than from the EMS or peer evaluation.

**Conclusion:**

In this single-site cohort using an internally developed simulation case, the QSAT provided MSF with excellent reliability. Self-assessment decreases the reliability of the MSF, and our data suggest self-assessment should not be a component of MSF. Use of the QSAT for MSF may be considered as a source of data for clinical competency committees.

## INTRODUCTION

An advantage of high-fidelity simulation is the provision of a variety of case presentations ranging from commonly-seen presentations to rare but critical pathologies while maintaining a clinical sense of urgency in a low-stakes environment.^1^ Simulation has evolved for formative and summative assessment.^2^ Assessment of residents in Emergency Medicine (EM) is required by the Accreditation Council for Graduate Medical Education (ACGME), and this has been codified with the release of the ACGME Milestones.^3^ Milestone guidelines recommend simulation as a means of assessment of EM residents for milestones 1–11 and 16–23.^3^

The Queen’s Simulation Assessment Tool (QSAT) was developed and subsequently validated in a multicenter study using EM residents, with the distinct purpose of using simulation as a means of assessment of resident performance in resuscitation.^4^,^5^ The QSAT displayed its ability to discriminate between junior and more-senior residents in performance in several case types, with senior residents consistently performing better in all but one of 10 case types previously measured. The authors also studied the use of the QSAT tool in a formalized Objective Structural Clinical Examination to be used for assessment within their residency program.^6^

Another ACGME-recommended means of EM resident assessment is multisource feedback (MSF). MSF is recommended by the ACGME to assess 10 of the 23 milestones.^3^ MSF would then be forwarded to the residency programs’ clinical competency committees (CCC), which would use the data as part of their process to determine milestone progression during semi-annual resident evaluation.

A relatively unexplored area of research in the use of simulation is the addition of other evaluating parties in a MSF, or 360-assessment model. Outside of the simulation environment, the feasibility and reliability of MSF within EM has been demonstrated.^7^ Here the instrument was more complex than the QSAT. Using similar questionnaire methodology, one study noted that MSF may bias toward favorable responses for physicians.^8^ Systematic review of several MSF studies shows adequate reliability with only eight coworkers or eight medical colleagues when surveyed.^9^ MSF is listed among potential options for evaluating residents for various ACGME core competencies.^3^

There is limited study of the use of MSF for resident assessment. A previous small trial with 10 residents assessed showed acceptable inter-rater reliability involving 44 nurse evaluations and 13 faculty evaluations. The trial demonstrated good interclass reliability between faculty and nurse assessments;^10^ however, that occurred with assessment of resident performance over several non-standardized cases.^10^ To date, little has been published on MSF evaluation of residents in general and in the simulation lab in particular. Our study sought to determine the concordance of rater evaluations of the QSAT assessment tool when used in MSF to assess EM resident simulation performance in a standardized, adult-simulation resuscitation performed in a simulation center setting.

**Population Health Research Capsule**

What do we already know about this issue?

*In the toolbox of suggested types of resident evaluation offered for Emergency Medicine (EM) Milestones, the Accreditation Council for Graduate Medical Education includes Multi-Source Feedback (MSF). MSF is often referred to as 360 feedback*.

What was the research question?

*This study sought to determine the degree of concordance of MSF using the Queen’s Simulation Assessment Tool (QSAT) in the simulation lab using a standardized adult resuscitation case*.

What was the major finding of the study?

*Compared to faculty evaluation as the gold standard, a peer resident, emergency medical services provider, and nurses provide concordant MSF with excellent inter-rater reliability. Self-evaluation was less reliable*.

How does this improve population health?

*This cohort suggests that the QSAT could be used to provide MSF data to EM Residency Clinical Competency Committees. The lower concordance suggests self-evaluation should not be a component of MSF*.

## METHODS

After institutional review board approval, we conducted this prospective study at a postgraduate year (PGY) 1–4 EM residency training 13 residents per year at a suburban healthcare network. The program is dually approved by both the ACGME and the American Osteopathic Association. All participants were consented prior to participation in the simulation cases, which were performed in the simulation lab during educationally protected grand rounds time. As part of the consent process, the contact information for an independent party at the hospital’s department of education was provided to each study participant. To further protect the participants, each had the ability to privately contact this independent party after the simulation to be anonymously removed from the study.

One designated adult Advanced Cardiac Life Support case was developed for this study by a panel of simulation-trained physicians using standard simulation templates (Appendix). All EM residents in their PGY 2–4 levels of training were eligible to be enrolled to serve as team leaders for the case. The team leader resident directed the simulation and resuscitation of the case and asked for telephone communication with consultant providers (toxicology and critical care) whenever appropriate. As team leader, he or she received MSF using a previously validated rubric. This instrument, the QSAT, was previously studied with attending physicians evaluating residents.^4^,^5^,^6^ The QSAT assesses resident performance on four factors of resuscitation leadership: primary assessment of the patient; initial diagnostic testing; treatment of the underlying condition; and interpersonal communication with staff and consultants. There is a fifth and final overall performance category. These aspects are rated on a 1–5 Likert scale, with a score of 1 representing delayed or incomplete performance of all aspects of care and 5 signifying competent performance of all aspects of care. The QSAT modified for this study simulation case is shown in Figure.

In this study, multiple healthcare staff members present during the performance of the case completed the QSAT. Two designated EM core teaching faculty (“faculty”) members, defined a priori as the gold standard, both completed a QSAT on each simulation. MSF was provided by nurses (RN), a resident peer (“peer”), and an emergency medical services (EMS) provider. As a PGY-1, the resident peer served as the junior resident for the enrolled team leader. The team leader (“self”) performed self-evaluation when completing the MSF. The QSAT was completed immediately upon conclusion of the simulation. The participants were not specifically trained on the QSAT. For the purposes of statistical analysis, the resident peer, the EMS provider, and the two faculty did not vary. All cases were performed using high-fidelity simulation mannequins that are age-appropriate for adult cases. We recorded demographics from all participants.

### Data Analysis

We used descriptive statistics to describe the sample, and counts and percentages to describe categorical variables. The mean and standard deviation was used to describe continuous variables found to be normally distributed, and we described non-normally distributed variables using the median. Normality was assessed by determining if the skew statistic was less than +1 and greater than -1 and upon visual inspection of a histogram plot. To avoid issues with repeated measures analysis secondary to unequal response rates, participating groups were either present for all simulations (defined as faculty, peer, EMS) or were enrolled for only one case (defined as self and RN).

The QSAT was cumulatively scored by adding the scores for each section, resulting in one total score ranging from 5–25. To test the hypothesis, we assessed inter-rater reliability (ie, the reliability of two or more raters measuring the same resident), by obtaining intraclass correlation coefficients (ICC) for the groups of raters. We used two-way random ICCs to determine the average level of absolute rater agreement between all raters within each simulation. Interpretation of the ICC was based on prior publication, with results less than 0.40 noted as poor, 0.40 to 0.59 fair, 0.60 to 0.74 good, and ≥ 0.75 excellent.^11^ We then calculated an ICC for the two attending physicians as one group with another ICC calculated for self, peer, RN, and EMS raters as a separate group. We also generated ICCs for the group as a whole after systematic removal and replacement of RN, peer, EMS, and self-raters from the whole group. An observer group was defined as the RN, peer resident, and EMS evaluators.

All analyses were two-tailed with alpha set at 0.05. We performed all statistical analyses using SAS version 9.3 (SAS Institute, Cary, NC) and SPSS version 24 (IBM SPSS Statistics for Windows, Armonk, New York). The study was supported by an unrestricted educational grant from the Dorothy Rider Pool Health Care Trust.

## RESULTS

We conducted four designated simulation sessions spanning six months. Thirty-four residents were enrolled as designated team leaders, 12 of whom were female (35.3%). The median age was 31. Twenty-five had a Doctor of Osteopathic Medicine (DO) degree (73.5%) with the remaining having a Doctor of Medicine (MD) degree; one participant (2.9%) held another advanced degree (Master in Business Administration). Nine residents were sampled at the end of their PGY-4 years (26.5%), 11 at the start of their PGY-4 years (32.4%), 10 during the start of their PGY-3 years (29.4%), and four at the start of their PGY-2 years (11.8%).

We used 34 different nurse-raters during the study; 30 were female (88.2%). The median number of years of experience was 4.5. Ten (32.3%) were nurses in their first year, enrolled in the healthcare network’s “nursing residency.” The remaining 21 nurses (67.7%) were recruited from the emergency department and had 9.5 median years of experience (IQR 4.5 -10.0). The median age of the nurses was 28.5. Most held Bachelor of Science degrees (82.4%), while three (8.8%) had another advanced degree (Master of Science). The EMS, peer resident and faculty raters were all male. Their experience was 13 years of EMS, PGY-1 level of training, and for faculty 14 and 15 years, respectively,..

The QSAT score averages and cumulative totals for resident team leaders in each category as rated by the evaluators are presented in Table 1. Self-evaluation scores were the lowest in all categories. Attending scores tended to be the highest in each category, with a few exceptions. The average total QSAT score for the self-evaluator was nearly 3.5 points lower than the total averaged score between the two attending evaluators. Remaining evaluators provided similar total scores as compared to the attending evaluators for the residents as a whole.

Total QSAT scores for individual residency levels were prepared in subgroups by PGY level of training (Table 2). The trend of lower total QSAT scores amid resident self-evaluation remained at all PGY levels. Total scores were high for all residents despite the PGY level. Despite differences in their levels of training, resident team leaders each performed very similarly according to each type of evaluators.

The ICCs for total QSAT scores are shown in Table 3. The ICCs for the inter-rater reliability of all raters across residents evaluated showed excellent correlation, with an ICC of 0.754 including all groups. ICCs for a group of observers including the RN, EMS provider and peer evaluator were calculated to be 0.806 (0.660–0.897) for inter-rater reliability.

We also calculated subgroup ICCs with individual categories of raters removed systematically (Table 3). The ICCs for inter-rater reliability were similar no matter what groups were removed, and 95% confidence intervals (CI) for all subgroups overlapped, showing no statistically significant difference. The lone exception was in the subgroup in which the self-evaluators were removed. Inter-rater ICCs increased markedly, although no statistically significant difference was shown between this and the overall ICCs with all groups.

We also compared ICCs of individual types of healthcare provider rater groups to each other (Table 4). The two attending physicians showed excellent inter-rater reliability with each other. When comparing the attending physicians to other rater groups, the least agreement was noted between attendings and nurses, while the strongest agreement came between attendings and the EMS provider. With 10 (32.3%) of enrolled nurses coming from the nursing training program, this agreement was explored further. Nurse residents had higher ICC inter-rater with the attendings (.680, .093-.913) than the more experienced nurses (.649, 0.300-.843). Comparing attendings to an observer group (RN, EMS and peer) showed good agreement in inter-rater reliability. No statistically significant difference was noted between any of these subgroup analyses, as all 95% CIs overlapped.

## DISCUSSION

In this study, all raters using the QSAT to assess performance on a standardized adult simulation case provided scores with excellent inter-rater reliability. Given that inter-rater reliability, or the ability to have one source of feedback agree with another, was excellent in this cohort suggests that the QSAT may be a viable instrument for MSF. Prior research has suggested that at least 30 measures from at least three raters should be used to calculate ICCs.^12^ This cohort met both of these criteria, lending further support to this finding.

The inter-rater reliability improved when self-assessment scores were removed. This is reflected in the raw data, which showed significantly lower self-rated evaluations compared to the other groups. Prior study of EM resident self-assessment in the simulation lab demonstrated variability in the accuracy of assessment as compared to attendings.^13^ This study found that agreement with attending evaluation increased with increasing attending scores. In general, physician self-assessment has been demonstrated to be of limited value.^14^ In this systematic review, as compared to objective measures, self-assessment has a wide range of variability. This cohort suggests that when using the QSAT, MSF should not include self-assessment. It may be reasonable to extend that conclusion to MSF more broadly.

When evaluating the specific sources of MSF in this cohort, the agreement between the two faculty evaluators was the highest. To add to this traditional source of resident feedback, the addition of EMS feedback performed the best. The EMS provider in this study helps run the healthcare network simulation lab, as well as teaching and assessing performance in life support classes to a range of providers including physicians. As such, the performance of EMS MSF in this cohort may not be generalizable.

The performance of the peer evaluator was very similar to that of the EMS provider, achieving excellent inter-rater agreement with the faculty. (The participation of the single peer began at the end of his PGY-1 year, and the study was completed during the first half of his PGY-2 year.) In contrast, the agreement of the RN evaluators had the lowest agreement with the faculty. This agreement actually decreased with increasing years of experience. This finding may suggest there are fundamental aspects of training and experience, which increases agreement for resident sources of MSF but decreases it for nursing. Differences in the evaluation of resident performance by physicians and nurses have been previously demonstrated.^15^ This finding may have implications for the inclusion of nursing in MSF moving forward. Alternatively, since both faculty evaluators as well as the peer and EMS evaluators were male, and the nurses overwhelmingly female (88.2%), another possible explanation for the differences in agreement is that gender may play a role. Previous study of the role of gender of faculty and residents as it relates to resident evaluation in internal medicine has not been conclusive.^16^,^17^ In EM specifically, the gender of the resident being evaluated has been shown to influence milestone evaluations by faculty.^18^

To determine if MSF from other healthcare providers could replace faculty evaluations, we created an observer group. This group comprised the peer, EMS, and RN evaluators. The inter-rater reliability of this group independently was excellent (.806). While the group did have excellent agreement among themselves, agreement with the faculty did not perform as well (.680). Having defined a priori that the faculty scores defined the gold standard, this suggests that attending input should consistently be a component of MSF.

Regarding individual QSAT categories, the one receiving the lowest overall score was “diagnostic testing.” The categories of “primary assessment” and “therapeutic actions” were the most highly rated among the evaluators. This may be the result of the specifics of the case, the qualities of the training program, the attributes of the residents enrolled, or a combination of the three. The scoring of the residents by PGY level did not demonstrate significant differentiation with increasing experience. This lack of heterogeneity may have impacted the calculation of the ICCs.^12^ Prior studies have demonstrated the ability of the QSAT differentiate resident performance.^6^,^19^ The inability to discriminate between residents in this cohort as they progressed, therefore, may be the result of the simulation case.

The chosen gold standard of a two-attending evaluation for the study is based on use of multiple attending physicians in previous QSAT studies. The agreement between the two faculty in our study was excellent (.840). However, one explanation for this high inter-rater reliability from the attending physicians could be due to bias resulting from their prior experience as faculty in the residency program. The original QSAT studies used independent, attending physician raters who were not faculty at the residents’ sites in order to minimize bias from familiarity with residents.^6^ For reasons related to availability, we used simulation-trained EM attending physicians who were known faculty; however, this could have led to them scoring residents highly due to their previous experience with these residents. While this may limit the results, it likely represents the manner in which the QSAT would be used by residency programs to gather MSF. This may increase the external validity of the study.

## LIMITATIONS

We performed this study at a single site. The details of the resuscitation case were developed internally and not validated, which may explain the observed inability to discriminate between more- junior vs more-senior residents. To avoid issues with repeated measures analysis, some of the sources of MSF were fixed to specific individuals, while other sources were random. The residents running the case were known to the faculty evaluators, which may have increased the scores provided. The sampling of resident team leaders was by convenience; and to ensure that the peer evaluator was junior to the team leader, we enrolled no PGY 1 residents in that role. Participants did not receive training on the use of the QSAT in an attempt to have the study reflect how the QSAT would likely be used for MSF. This lack of training may have impacted the findings.

## CONCLUSION

In this single-site cohort using an internally developed, standardized adult simulation case, we found that the QSAT can provide MSF with excellent interrater reliability. EM residency CCCs may consider using the QSAT to provide ACGME-recommended multi-source feedback. Nurses as a group had lower inter-rater reliability than other evaluators present during the case, which may have been the result of training or gender, or both. Self-evaluation should not be a component of MSF given that this cohort demonstrated the lowest inter-rater reliability.

## Supplementary Material



## Figures and Tables

**Figure:**
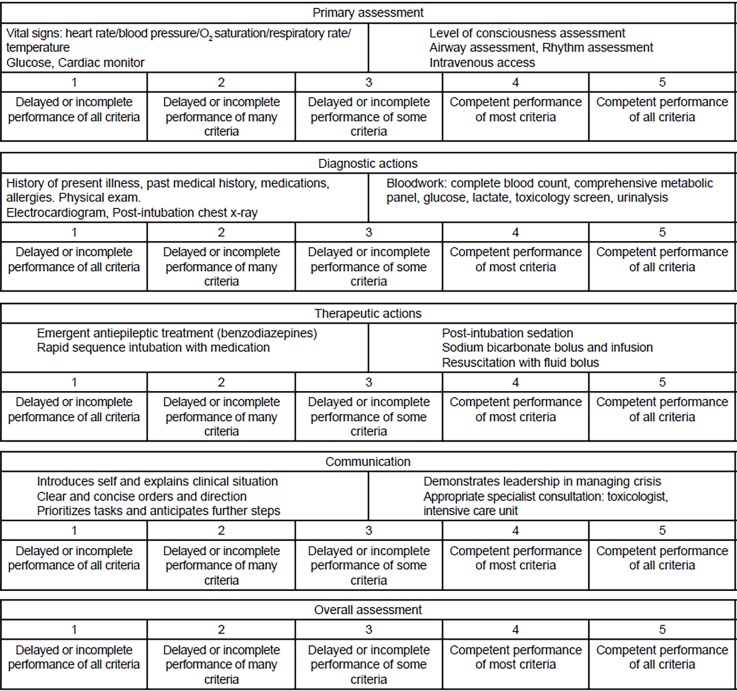
Modified Queen’s simulation assessment tool.

**Table 1:** QSAT Likert scores for resident evaluation for individual categories.

	Primary assessment mean (SD)	Diagnostic actions mean (SD)	Therapeutic actions mean ((SD)	Inter-personal communication mean (SD)	Overall assessment mean (SD)	Total QSAT scores mean (SD)
Self	4.06 (0.49)	3.79 (0.69)	4.06 (0.81)	4.09 (0.67)	3.88 (0.59)	19.88 (2.58)
Peer	4.79 (0.48)	4.18 (0.80)	4.26 (0.96)	4.62 (0.55)	4.38 (0.65)	22.24 (2.69)
Nurse	4.56 (0.50)	4.29 (0.68)	4.62 (0.60)	4.68 (0.59)	4.41 (0.50)	22.56 (1.93)
EMS	4.76 (0.50)	4.41 (0.61)	4.47 (0.71)	4.71 (0.58)	4.50 (0.75)	22.85 (2.63)
Attg 1	4.88 (0.33)	4.62 (0.49)	4.50 (0.66)	4.88 (0.41)	4.74 (0.45)	23.62 (1.56)
Attg 2	4.94 (0.24)	4.56 (0.66)	4.12 (0.98)	4.88 (0.33)	4.47 (0.79)	22.97 (2.11)

*QSAT*, Queen’s simulation assessment tool; *Attg*, attending physician; *SD*, standard deviation; *EMS*, emergency medical services.

**Table 2:** Total QSAT scores for resident evaluation by PGY year.

PGY Year	Evaluator	Score; mean (SD)
End of PGY-4	Self	19.44 (1.74)
	Peer	21.00 (3.57)
	Nurse	21.89 (1.69)
	EMS	22.11 (2.71)
	Attending 1	23.67 (1.00)
	Attending 2	23.44 (1.24)
End of PGY-3 / Start of PGY-4	Self	19.55 (2.66)
	Peer	23.00 (2.32)
	Nurse	22.45 (2.21)
	EMS	22.55 (3.17)
	Attending 1	24.00 (1.79)
	Attending 2	23.27 (2.24)
End of PGY-2 / Start of PGY-3	Self	20.90 (2.96)
	Peer	22.00 (2.26)
	Nurse	22.70 (1.95)
	EMS	23.90 (2.18)
	Attending 1	23.00 (1.89)
	Attending 2	22.60 (2.76)
Start of PGY-2	Self	19.25 (3.20)
	Peer	23.50 (1.73)
	Nurse	24.00 (1.15)
	EMS	22.75 (1.71)
	Attending 1	24.00 (0.82)
	Attending 2	22.00 (1.63)

*QSAT*, Queen’s simulation assessment tool; *PGY*, postgraduate year; *SD*, standard deviation; *EMS*, emergency medical services.

**Table 3:** Interrater reliability by intraclass correlation coefficients for total QSAT scores with 95% confidence intervals .

All raters	Self removed	Peer removed	Nurses removed	EMS removed	Attendings removed
0.754 (0.572–0.867)	0.838 (0.733–0.910)	0.667 (0.412–0.822)	0.715 (0.484–0.850)	0.660 (0.408–0.817)	0.680 (0.423–0.831)

*QSAT*, Queen’s simulation assessment tool; *EMS*, emergency medical services.

**Table 4:** Intraclass correlation coefficients between attending physicians and other healthcare providers for total QSAT score with 95% confidence interval.

	Attendings only	Attendings + observers	Attendings + peer	Attendings + nurse	Attendings + EMS
Interrater reliability	0.840 (0.634–0.925)	0.680 (0.423–0.831)	0.779 (0.594–0.885)	0.651 (0.394–0.812)	0.812 (0.670–0.900)

*QSAT*, Queen’s simulation assessment tool; *EMS*, emergency medical services.
