# Supplementary material for: Assessment of Emergency Medicine Resident Performance in an Adult Simulation Using a Multisource Feedback Approach
Source: West J Emerg Med. 2018 Dec 17;20(1):64–70. doi: 10.5811/westjem.2018.12.39844 (PMC6324708; doi:10.5811/westjem.2018.12.39844)
Supplement: Supplementary file 1 [file wjem-20-64-s001.docx]

**Appendix.** Tricyclic Antidepressant Overdose - 360 Case.

**1. Objectives:**

1. Lead a treatment team through the resuscitation of a tricyclic antidepressant (TCA) overdose based on patient presentation, ECG findings, and metabolic abnormalities
2. Demonstrate competency in the following areas as determined by the Queen’s Simulation Assessment Tool
   1. Performance of primary survey (PC1)
   2. Performance of appropriate diagnostic actions:
      1. Obtaining a history and physical exam (PC2)
      2. Obtaining and interpreting appropriate diagnostic studies (PC3)
   3. Communicate effectively with:
      1. Treatment team (ISC2)
      2. Consultants (ISC2)

**2.**     **Learners:**

1. Emergency medicine residents beyond the PGY-1 year
   1. To evaluate function as team leader
   2. To be subject of formative assessment

**3.**     **Location:**

1. LVHN simulation center

**4.**     **Patient:**

1. 36-year-old male who presents with active seizure. Patient was discovered this way by a friend

**5.**     **Equipment:**

1. Simulation manikin (i.e. SimMan)
2. IO trainer
3. Oxygen non-rebreather mask
4. Bag-valve-mask
5. Nasal cannula
6. Intubation equipment
7. EZ-IO
8. Supplies for IV access
9. Saline filled syringes to simulate medications
10. Cardiac monitor
11. Defibrillator

**6.**     **Moulage:**

1. Patient will initially present with seizure activity, represented by shaking the bed.
2. After seizure activity ceases, patient will be minimally responsive, groaning, GCS card will reveal following:
   1. Does not move extremities to stimuli
   2. Nonverbal
   3. Eyes closed despite painful stimuli
3. Card tag on patient’s skin underneath clothing saying “Skin: warm and dry”

**7.**     **Confederates:**

1. EMS personnel to provide initial history of case
   1. Patient was found by friend to be in seizure activity for several minutes, was not responding to anything. Friend reports that he last saw him well last night when he went home from the bar. Friend called 911, EMS uncertain if he is on the way. They just “scooped and ran.” Provided 2 mg IV Lorazepam en route which did not resolve symptoms.
   2. IF PROMPTED: EMS found several pill bottles scattered around (produces pill bottles). Also, EMS did notice a note next to the patient, which he/she did not scan thoroughly (produces suicide note).
2. Nurse resident
3. Faculty/technician to operate manikin
4. Designated PGY-1 resident serving as team member
5. Faculty assessors

**8.**     **Supporting Files:**

1. EKG: sinus tachycardia, wide QRS with terminal R-wave in lead aVR, QT prolongation
2. Point-of-care glucose: 110
3. CXR image: post intubation, no other abnormalities
4. Lab handouts
   1. CBC (unremarkable)
   2. CMP (notable for bicarbonate 10, no anion gap)
   3. VBG/ABG (metabolic acidosis pH 7.10 )
   4. UA (unremarkable)
   5. Other labs only available if specifically asked for (ASA, APAP, RUDS, lactate, etc)

**9.**     **Case Synopsis:**

1. This patient will require resuscitation with eventual intubation and treatment for underlying cause of disease (TCA overdose). The goal of the scenario is for the assigned leader to direct the team to perform all actions required
2. Attention should be paid to:
   1. The leader addressing everyone on the team
   2. The leader incorporating team members’ suggestions
   3. The team closing communication loops
      1. For high-complexity decisions like medical dosing
      2. To confirm actions are performed
3. Faculty should try and run scenario in real-time as much as possible
   1. Avoid artificially “fast-forwarding” through case
   2. Do not consider an action performed until nursing confirms completion of action (i.e. medication given)

**10.**     **Case Timeline / Flow:**

1. Initial condition:
   1. Settings
      1. Temp 100.2 F
      2. HR 142
      3. BP 82/46
      4. RR 22
      5. SpO2 95%
      6. Rhythm: RBBB on monitor
   2. Seizure activity (shaking the bed)
   3. Actions required:
      1. Establish IV/IO access
      2. Cardiac monitoring
      3. Supplementary oxygen with NC
      4. Point-of-care glucose
      5. IV fluid bolus 1-2 L crystalloid solution
      6. Benzodiazepine at appropriate dosage for seizure treatment (i.e. Lorazepam 2 mg IV)
2. Post-seizure to post-intubation
   1. Settings
      1. HR 120
      2. BP 90/50
      3. Vitals otherwise unchanged
      4. Rhythm: RBBB on monitor
   2. Patient minimally responsive
   3. Actions required:
      1. Intubation with RSI
      2. Appropriate post-intubation sedation
      3. EKG
      4. Post-intubation CXR
      5. Labs
      6. Recognition of EKG pattern and/or history consistent with TCA overdose
      7. Appropriate treatment with sodium bicarbonate 50 mEQ bolus + NaHCO3 infusion
3. Post-NaHCO3 treatment
   1. Settings
      1. HR 110
      2. BP 105/57
      3. Rhythm: Sinus tachycardia on monitor
   2. Actions required
      1. Discussion with toxicologist
      2. Discussion with intensivist for admission
4. Complications
   1. If TCA overdose not recognized or improperly treated during Stage B (post-seizure to post-intubation), patient can devolve to stage A (initial condition)
   2. If intubation equipment (i.e. Laryngoscopes blade) placed in oropharynx without RSI medications, have the patient vomit.

**11.     Assessment Tools**

1. Two assessment tools:
   1. Queen’s Simulation Assessment Tool (QSAT)
   2. To be completed by healthcare workers in multiple disciplines
      1. Resident learner (self-assessment)
      2. Two emergency medicine residency faculty members
      3. Another resident who was a part of the resuscitation team
      4. Nursing resident who served as nurse for the case
      5. EMS provider
2. Evaluators should pay attention to the following actions when completing the assessment tool:
   1. Emergency Stabilization (PC1)
      1. Performance of primary survey
         1. Verbalize an airway assessment
         2. Listen to the lungs
         3. Feel for a pulse
      2. Establishment of safety net
         1. IV/IO access
         2. O2
         3. Monitor
         4. Point-of-care glucose
   2. Performance of a focused history and physical examination (PC2)
      1. Obtain AMPLE history as possible from EMS
      2. Exposes and rolls patient
   3. Diagnostic studies (PC3)
      1. EKG
      2. CXR
      3. POC glucose
      4. Labs
   4. Pharmacotherapy (PC5)
      1. Emergent antiepileptic therapy with IV benzodiazepine
      2. Sodium bicarbonate treatment for TCA overdose
      3. RSI for intubation
   5. Airway management (PC10)
      1. Supplementary O2 by nasal cannula
      2. Intubation with RSI
   6. Team management (ICS2)
      1. Team leadership
      2. Consultation with toxicologist
      3. Handoff of care to intensivist
